# Novel MRI‐Based Pedicle Bone Quality Score Independently Predicts Pedicle Screw Loosening after Degenerative Lumbar Fusion Surgery

**DOI:** 10.1111/os.14146

**Published:** 2024-07-09

**Authors:** Qiujiang Li, Haiying Fu, Huiliang Yang, Xi Yang, Lei Wang, Yueming Song

**Affiliations:** ^1^ Department of Orthopedic Surgery, West China Hospital Sichuan University Chengdu People's Republic of China; ^2^ Department of Orthopedic Surgery, West China Hospital, Sichuan University/West China School of Nursing Sichuan University Chengdu China

**Keywords:** Bone Quality, Hounsfield Unit, MRI, Pedicle Bone Quality, Screw Loosening, Vertebral Bone Quality

## Abstract

**Summary:**

Pedicle screw loosening after posterior lumbar fusion is associated with poor bone quality, which often determines screw pull‐out strength, insertion torque, and vertebral body loading characteristics. Magnetic resonance imaging (MRI)‐based vertebral bone quality (VBQ) score were associated with poor bone quality. Current evidence suggests that pedicle bone quality (PBQ) has a greater impact on screw stability. However, the correlation between MRI‐based PBQ score and screw loosening has not been reported.

**Purpose:**

To introduce and evaluate an MRI‐based PBQ score to determine its effectiveness in predicting pedicle screw loosening following lumbar fusion surgery.

**Methods:**

The retrospective study analyzed 244 patients who underwent posterior lumbar interbody fusion (PLIF) with pedicle screws between December 2017 and December 2021, with CT and MRI imaging before surgery. Data collected included patient demographics and preoperative radiological data. Radiographic screw loosening was measured at 12 months postoperatively. Clinical assessments included pain visual analog scale (VAS) and Oswestry Disability Index (ODI) scores. The PBQ score was measured using MRI scans. We use univariate analysis for preliminary screening of the risk factors of screw loosening. Subsequent analysis involved multivariate logistic regression to identify independent predictive factors for screw loosening. We constructed the receiver operating characteristic (ROC) curve to ascertain the discriminative capacity of the PBQ score. The area under the curve (AUC) quantified its predictive accuracy. Additionally, we evaluated the association between PBQ score and screw loosening using Spearman's correlation analysis.

**Results:**

Overall, 244 patients who underwent PLIF with pedicle screw fixation participated in this study, including 35 in the loosening group and 209 in the non‐loosening group. PBQ score in the loosening group was significantly higher than that in the non‐loosening group. On multivariate logistic regression, the higher PBQ score (OR = 8.481, 95% CI: 3.158–22.774; *p* < 0.001) and the lower mean Hounsfield unit (HU) value of L1‐4 (OR = 0.967, 95% CI 0.951–0.984; *p* < 0.001) were the variables that significantly predicted screw loosening. The AUC for the PBQ score and HU value were 0.751 (95% CI: 0.673–0.828) and 0.702 (95% CI: 0.612–0.791). The PBQ score optimal cutoff to differentiate patients with loosening and with non‐loosening was calculated as 3.045 with a sensitivity of 85.7% and specificity of 76.9%, while the optimal cutoff of the HU value was 151.5 with a sensitivity of 64.6% and specificity of 89.5%.

**Conclusions:**

The association between the PBQ score and the propensity for lumbar pedicle screw loosening was found to be substantial. As a predictive measure, the PBQ score outperformed the HU value in forecasting the likelihood of screw loosening post‐posterior lumbar fusion.

## Graphical abstract

The findings of this study suggest that PBQ score was significantly associated with lumbar pedicle screw loosening. PBQ score was a better predictor of pedicle screw loosening after posterior lumbar fusion than HU value.

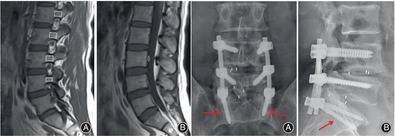

## Introduction

Pedicle screw loosening represents a frequent complication after lumbar fusion surgeries.^1^, ^2^, ^3^ This issue may give rise to additional complications including screw breakage, lack of spinal bone fusion, formation of false joints, and abnormal spinal curvature. Such events can compromise spinal integrity, potentially causing discomfort and neurological impairments.^4^, ^5^, ^6^ Consequently, preventing screw loosening is of considerable importance.

In patients undergoing instrumented spinal fusion, bone quality generally determines screw pullout strength, insertion torque, and vertebral body loading characteristics.^7^, ^8^ Therefore, preoperative bone mineral density (BMD) assessment is critical to surgical planning. The current gold standard for assessing BMD is dual energy X‐ray absorptiometry (DEXA).^9^, ^10^ Nonetheless, this method's exactitude is compromised in the presence of conditions such as vascular calcification, obesity, degenerative afflictions of the lumbar region, or antecedent spinal operations, compounded by its incapacity to appraise bone mineral density at precise localities, notably within pedicular structures.^11^, ^12^, ^13^ Quantitative computed tomography (QCT) and MRI‐based assessments of bone quality have emerged as superior alternatives for the determination of BMD, particularly within the lumbar spine. Both MRI‐based VBQ score and CT‐based Hounsfield unit (HU) value can achieve accurate screw loosening prediction.^4^, ^6^, ^7^ MRI holds a distinct benefit over its counterparts by eschewing radiation exposure, thus shaping it as a routine, non‐invasive preoperative assessment prior to engaging in spinal surgical procedures.

Recently, magnetic resonance imaging (MRI) based vertebral bone quality (VBQ) score has emerged as a new technique for assessing bone quality.^14^, ^15^, ^16^, ^17^, ^18^, ^19^ VBQ score can reflect the degree of marrow fat infiltration in lumbar vertebral medulla. Previous studies have also demonstrated that VBQ score predicts pedicle screw loosening after lumbar fusion.^20^ Therefore, MRI‐based bone quality score is a simple, effective, and reliable tool for predicting screw loosening. Previous studies have found that vertebral body and pedicle BMD were closely related to fixation stability.^7^ Studies have shown that pedicle BMD has a greater impact on screw stability than vertebral BMD.^21^, ^22^ Therefore, preoperative prediction of the initial fixation strength of pedicle screws is helpful in making surgical decisions and reducing surgical failure rates.^21^, ^23^ Therefore, in the present study, we (i) attempted to create a simple MRI‐based score to assess pedicle bone quality and (ii) investigated possible associations between pedicle bone quality (PBQ) score and pedicle screw loosening after degenerative lumbar fusion surgery.

## Methods

### Patient Population

We included 244 patients who underwent posterior lumbar interbody fusion (PLIF) with pedicle screw fixation for degenerative lumbar disease between December 2017 and December 2021. All patients included in the study underwent surgery by the same spinal surgery team. Allograft bone was used as fixation material in all patients, and bone cement was used in none of the patients. On the first postoperative day, the patient walked with a lumbar brace (lumbosacral orthosis or thoracolumbosacral orthosis) and wore the brace for 3 months and performed only essential daily activities. Institutional review board approval was secured for this study (no. 2021223), with all participants providing written informed consent. Inclusion criteria were as follows: (1) patients who underwent PLIF with pedicle screw fixation for degenerative lumbar disease; (2) age ≥ 50 years; (3) simultaneous lumbar MRI and CT scans within 1 week of surgery; and (3) no history of spine surgery. Exclusion criteria included the following: (1) the presence of scoliosis; (2) localized sclerosis of vertebral cancellous bone; (3) history of vertebral fracture or neoplasm; (4) metabolic and hematopoietic disorders other than osteoporosis; (5) levels of fixation >4 and lowest instrumented vertebra (LIV) higher than L4 level; (6) intraoperative adjustment of screw position and even pedicle wall damage during screw insertion; and (7) lacking complete clinical follow up data more than 1 year after surgery. Data collected included patient demographics and preoperative radiological data. Demographic data include age, gender, body mass index (BMI), smoking history, alcohol history, diabetes, hypertension, etc. Radiological data included the mean HU value of L1‐4, and lumbar T score based on DEXA, as well as pedicle bone quality (PBQ) score measured on T1‐weighted MRI images.

### PBQ Measurements

**FIGURE 1:**
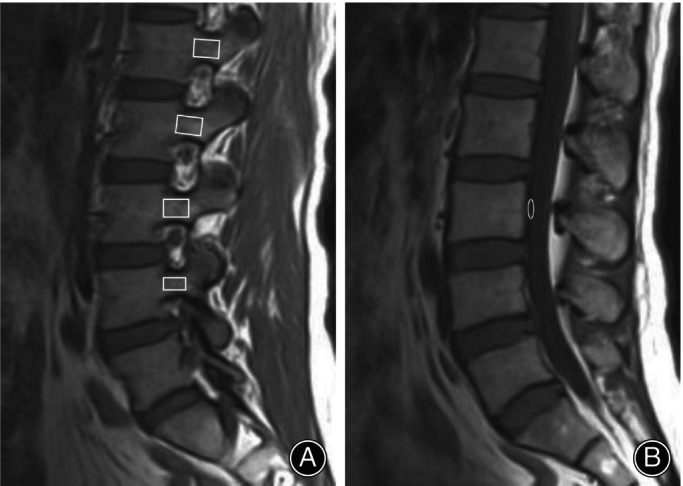
Representative image of ROI used to calculate the PBQ score. (A) ROI of pedicle; (B) ROI of CSF.

PBQ score refers to vertebral bone quality (VBQ) score measurements previously described by Ehresman et al.^24^ We measured PBQ score utilizing T1‐weighted sagittal MRI delineations specific to lumbar sites. As shown in Figure 1, the vertebral ROI was placed 1 mm from the pedicle cortex perimeter. Special care was taken to exclude any focal lesions, and if all sagittal sections were affected, PBQ score was derived exclusively from the mean values associated with the pedicles at the unaffected strata. A cerebrospinal fluid (CSF) ROI was placed in the posterior L3 vertebral body in an area without descending nerve roots. If CSF was affected at the L3 level, CSF measurements at the L2 or L4 level were selected. The PBQ score was then calculated by dividing the median signal intensity (SI) of the L1‐L4 pedicles by the SI of the CSF at the L3 level. The definitive PBQ score reflected the averaged bone quality indices extracted from both pedicles. All imaging parameters were quantified by a pair of surgeons extraneous to the primary procedure and the resultant analyses incorporated the mean derived from both sets of measurements.

### HU Value Measurements

**FIGURE 2:**
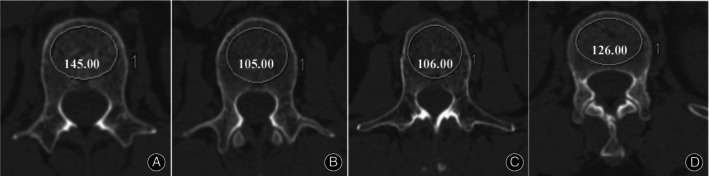
Example HU values of L1 (A), L2 (B), L3 (C), and L4 (D).

All patients underwent lumbar CT examination before operation. As shown in Figure 2, vertebral body HU was measured by drawing as large an elliptical region of interest (ROI) as possible within the cancellous bone on CT axis. ROI includes as much trabecular bone as possible and avoids cortical bone and heterogeneous regions such as posterior venous plexus, bone islands, and compressed bone. The average value of the upper, middle, and lower planes of each vertebral body was taken as the HU value of the vertebral body. Finally, the mean HU value of L1‐4 was used to represent the overall bone mineral density (BMD) of the lumbar. Same as PBQ, all imaging parameters were quantified by a pair of surgeons extraneous to the primary procedure and the resultant analyses incorporated the mean derived from both sets of measurements.

### Clinical Assessments

**FIGURE 3:**
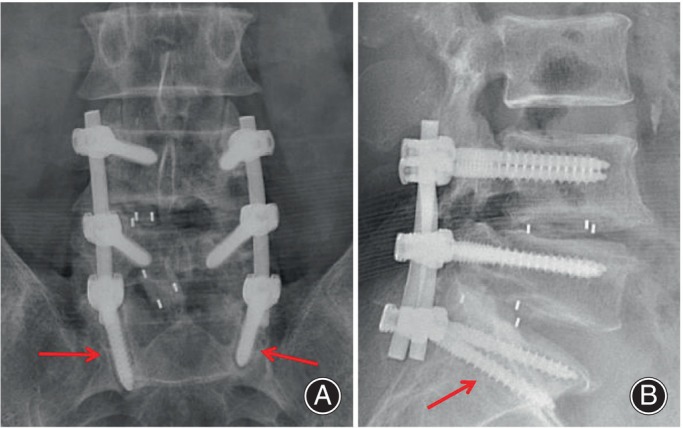
Pedicle screw loosening on anteroposterior (A) and lateral (B) radiographs. The radiolucent line indicated by red arrow.

Radiological examinations were performed at 1 week, 3 months, 6 months, and 1 year after operation. The evaluation of post‐surgical screw instability was principally predicated upon lumbar radiography. Contingent upon necessity, an ancillary computed tomography (CT) scan was procured. The criterion for diagnosing screw instability was the presence of a translucent halo of at least 1 ml thickness encircling the pedicle screw on radiographic images (Figure 3). At the 1‐year postoperative mark, patients were stratified into two cohorts: one exhibiting screw instability and the other remaining stable. To appraise clinical outcomes, metrics such as the pain visual analog scale (VAS) and the Oswestry Disability Index (ODI) were obtained from all subjects both prior to surgery and during the 1‐year follow‐up interval. The acquisition of follow‐up data for all participants was facilitated through either direct outpatient consultations or telephonic outreach.

### Inter‐Observer Agreement

We randomly selected 50 patients among all patients for interobserver and intraobserver agreements testing. Radiographic parameters were measured by two surgeons who had not participated in the primary surgery. The inter‐observer agreements were assessed using the intra‐class correlation coefficient (ICC), and wherein an ICC greater than or equal to 0.75 was indicative of commendable reliability. All radiographic metrics were analyzed by computing the average of the two readings.

### Statistical Analysis

For the assessment of continuous variables, independent t‐tests or analyses of variance were employed, while categorical variables underwent examination through the chi‐square test or Fisher's exact test. Preliminary scrutiny of risk factors associated with screw loosening was conducted using univariate analysis. Subsequently, we advanced to multivariate logistic regression analysis to sift through and identify independent risk determinants. In our quest to distinguish the separation parameters for groups unaffected by loosening versus those compromised by it, a receiver operating characteristic (ROC) curve analysis was undertaken, and the area under the curve (AUC) was computed. The ROC curve facilitated the establishment of a PBQ score threshold that possessed elevated levels of sensitivity and specificity. The association between PBQ score and the incidence of screw loosening was scrutinized using the Spearman correlation test. For all statistical computations, the Statistical Package for the Social Sciences (SPSS Inc., Chicago, IL, USA) was employed. Disparities were deemed statistically significant when p‐values fell below the 0.05 threshold.

## Results

### Demographics

Overall, 244 patients who underwent PLIF with pedicle screw fixation participated in this study, including 35 in the loosening group and 209 in the non‐loosening group. The mean age for the whole cohort was 61.39 ± 7.92 years old, 156 (63.9%) were female and 88 (36.1%) were male. The rate of LIV at S1 was 54.3% (19/35) in the loosening group, which was significantly more than that in the non‐loosening group (35.4% [74/209]) (*p* = 0.033). When comparing the demographic and perioperative radiographic data which included age (*p* = 0.774), gender (*p* = 0.600), BMI (*p* = 0.627), diagnosis (*p* = 0.933), use of anti‐osteoporosis drugs (*p* = 0.182), levels of fixation (*p* = 0.471), and lumbar T‐score (*p* = 0.242) between the two groups, no significant differences were found (Tables 1 and 2). Other indicators of clinical outcome were not statistically different between the two groups (Table 3).

**TABLE 1:** Demographics and perioperative radiographic data for pedicle screw loosening.

Variable	Total (n = 244)	Loosening (n = 35)	Non‐loosening (n = 209)	*p* value
Age, mean ± SD	61.39 ± 7.92	61.03 ± 6.77	61.44 ± 8.11	0.774
Gender, N (%)				0.600
Male	88 (36.1)	14 (40.0)	74 (35.40)	
Female	156 (63.9)	21 (60.0)	135 (64.6)	
BMI, mean ± SD, kg/m^2^	25.23 ± 3.61	25.51 ± 3.04	25.19 ± 3.70	0.627
Smoker, N (%)				0.581
Yes	54 (22.1)	9 (25.7)	45 (21.5)	
No	190 (77.9)	26 (74.3)	164 (78.5)	
Alcohol abuse, N (%)				0.449
Yes	51 (20.9)	9 (25.7)	42 (20.1)	
No	193 (79.1)	26 (74.3)	167 (79.9)	
Hypertension, N (%)				0.878
Yes	74 (30.3)	11 (31.4)	63 (30.1)	
No	170 (69.7)	24 (68.6)	146 (69.9)	
Diabetes, N (%)				0.667
Yes	36 (14.8)	6 (17.1)	30 (14.4)	
No	208 (85.2)	29 (82.9)	179 (85.6)	
Diagnosis, N (%)				0.933
Lumbar stenosis	119 (48.8)	18 (51.4)	101 (48.3)	
Degenerative spondylolisthesis	64 (26.2)	9 (25.7)	55 (26.3)	
Spondylolytic spondylolisthesis	61 (25.0)	8 (22.9)	53 (25.4)	
Use of anti‐osteoporosis drugs, N (%)				0.182
Yes	109 (44.7)	12 (34.3)	97 (46.4)	
No	135 (55.3)	23 (65.7)	112 (53.6)	
Levels of fixation, mean ± SD	2.93 ± 0.87	3.03 ± 0.79	2.91 ± 0.88	0.471
LIV at S1, N (%)				0.033
Yes	93 (38.1)	19 (54.3)	74 (35.4)	
No	151 (61.9)	16 (45.7)	135 (64.6)	
Lumbar T‐score, mean ± SD	−1.42 ± 1.12	−1.62 ± 1.24	−1.38 ± 1.10	0.242
Mean HU value of L1‐4, mean ± SD	131.87 ± 26.96	113.46 ± 29.03	134.95 ± 25.40	<0.001
PBQ, mean (SD)	3.04 ± 0.55	2.97 ± 0.53	3.45 ± 0.46	<0.001

**TABLE 2:** Multivariate logistic regression analysis of risk factors for pedicle screw loosening.

Variable	Odds ratio	95% CI	*p* value
Age	1.006	0.954, 1.060	0.834
Gender	1.556	0.539, 4.495	0.414
BMI	1.08	0.954, 1.221	0.224
Smoker	1.226	0.351, 4.287	0.749
Alcohol abuse	0.653	0.231, 1.844	0.421
Hypertension	1.226	0.436, 3.449	0.699
Diabetes	0.763	0.230, 2.533	0.658
Use of anti‐osteoporosis drugs	2.618	0.957, 7.162	0.061
Levels of fixation	1.339	0.763, 2.351	0.308
LIV at S1	0.303	0.123, 0.747	0.009
Lumbar T‐score	0.781	0.526, 1.161	0.222
Mean HU value of L1‐4	0.967	0.951, 0.984	<0.001
PBQ	8.481	3.158, 22.774	<0.001

**TABLE 3:** VAS and ODI scores between the two groups.

Outcome	Loosening	Non‐loosening	*p* value
Preoperatively			
VAS back	6.20 ± 0.68	6.03 ± 0.94	0.315
VAS leg	5.54 ± 0.78	5.76 ± 0.88	0.169
ODI	37.63 ± 14.45	39.56 ± 15.06	0.480
Postoperative 12 m			
VAS back	2.23 ± 0.77	2.11 ± 0.86	0.446
VAS leg	1.80 ± 0.72	1.89 ± 0.93	0.538
ODI	12.83 ± 8.04	13.14 ± 8.07	0.831

### PBQ Score and Screw Loosening

**FIGURE 4:**
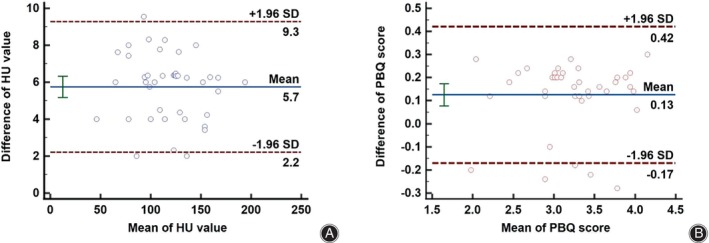
Bland–Altman plots of two authors for HU value (A) and PBQ score (B).

The HU value and PBQ score in 40 randomly selected patients were measured by two authors, which were in good agreement (ICC = 0.964, 0.910, respectively). The corresponding Bland–Altman plots are shown in Figure 4. There were significant differences between the two groups in mean HU value of L1‐4 and PBQ score (Table 1). PBQ score in the loosening group was significantly higher than that in the non‐loosening group (3.45 ± 0.46 *vs*. 2.97 ± 0.53, *p* < 0.001). Common risk factors were chosen as potential confounders for screw loosening and were included in the logistic regression analysis. On multivariate logistic regression, the higher PBQ score (OR = 8.481, 95% CI: 3.158–22.774; *p* < 0.001), the lower mean HU value of L1‐4 (OR = 0.967, 95% CI 0.951–0.984; *p* < 0.001), and LIV at S1 (OR = 0.303, 95% CI 0.123–0.747; *p* = 0.009) were the variables that significantly predicted screw loosening.

**FIGURE 5:**
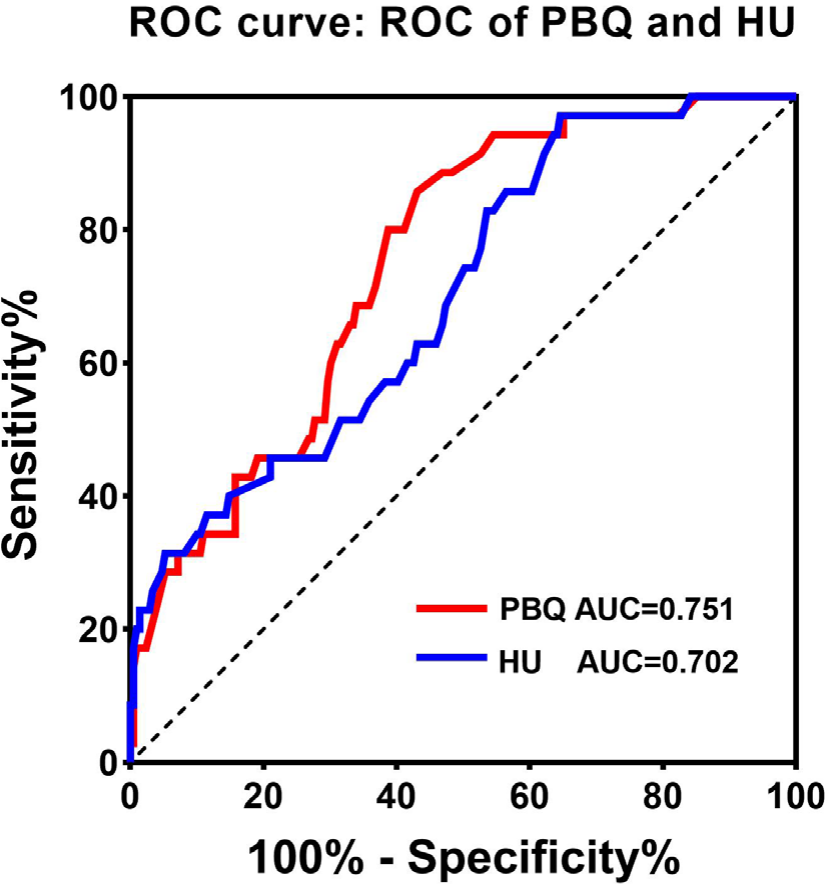
ROC curve analysis and AUC of HU value and PBQ score.

We established the ROC curve to evaluate the validity of using PBQ score and HU value to predict screw loosening (Figure 5). The AUC for the PBQ score and HU value were 0.751 (95% CI: 0.673–0.828) and 0.702 (95% CI: 0.612–0.791). The cutoff point was specified from the ROC curve using the optimal intersection of specificity and sensitivity. The PBQ score optimal cutoff to differentiate patients with loosening and with non‐loosening was calculated as 3.045 with a sensitivity of 85.7% and specificity of 76.9%, while the optimal cutoff of the HU value was 151.5 with a sensitivity of 64.6% and specificity of 89.5%. No significant correlation was observed between higher PBQ score and the investigated preoperative variables (Table 4).

**TABLE 4:** Univariate linear regression analysis for association between preoperative variables and pedicle bone quality score.

Variable	Estimate	Standard error	*p* value
Age	−0.006	0.004	0.203
Gender	0.103	0.081	0.207
BMI	−0.019	0.01	0.051
Smoker	0.081	0.095	0.397
Alcohol abuse	0.068	0.088	0.441
Hypertension	0.012	0.099	0.905
Diabetes	0.047	0.081	0.566
Diagnosis	0.044	0.045	0.320
Use of anti‐osteoporosis drugs	−0.062	0.072	0.390
Lumbar T‐score	0.022	0.032	0.480
Mean HU value of L1‐4	−0.002	0.001	0.065

## Discussion

In this study, we propose a new, MRI‐based bone quality score for specific pedicle sites. To the best of our knowledge, this is the first study to assess the correlation between MRI‐based PBQ score and pedicle screw loosening. The results showed that PBQ score was a risk factor for pedicle screw loosening after posterior lumbar fusion. The PBQ, like the mean HU value of the lumbar, is a valid predictor of screw loosening.

### Screw Loosening Rate

Screw loosening is one of the common complications after posterior spinal fusion.^2^, ^5^, ^23^ Screw loosening rates vary widely, depending on the study. Screw loosening was less than 10% for single‐level fixation and more than 40% for ≥4 levels.^25^ The results of this study showed a screw loosening rate of 16.74%, which may be related to the relatively short fixation segment of the population included in this study (2.93 ± 0.87). The primary purpose of pedicle screw fixation is to provide stability during spinal fusion to achieve spinal fusion. Pedicle screw loosening had an adverse effect on interbody fusion, pseudoarthrosis was present in more than half of cases, and was significantly associated with low back pain.^8^, ^20^, ^25^ This is confirmed by the higher VAS scores for low back pain we found in patients with screw loosening.

### Risk Factors for Screw Loosening

Risk factors for pedicle screw loosening have been widely reported, including advanced age, low BMD, multilevel fixation, and lowest instrumented vertebra (LIV) at S1.^2^, ^8^, ^20^ In multivariate analysis, LIV at S1, mean HU at L1‐4, and PBQ score were independent influencing factors for screw loosening. Age was not an independent risk factor. The reason is that old age is accompanied by bone mass decline, and age affects screw loosening through other media. There was no significant difference in T score between the two groups in this study. In this study, the average age was 61.39 ± 7.92 years old. A considerable part of the population had degenerative changes of lumbar spine, among which osteophyte formation, vertebral collapse, and compression fracture would mask the true bone mass level, resulting in false increase of BMD results.^26^, ^27^ Muraki et al.^27^ conducted a retrospective analysis of lumbar X‐ray findings in 630 women aged ≥60 years. The results showed that osteophyte formation, osteosclerosis, and disc stenosis scores were independently associated with lumbar BMD.

### The Levels of Fixation and Screw Loosening

The levels of fixation also had a significant effect on the rate of screw loosening. Studies have reported a screw loosening rates of less than 10% for one level fixation and more than 40% for ≥4 levels.^25^ Our results are consistent with previous studies, with a screw loosening rate of 14.34%. Our study sample size was relatively small and may not have sufficient statistical power to detect subtle differences between screw loosening and the number of fixation segments. Secondly, it is also related to the small number of fixed segments in this study. Therefore, we believe that further studies in larger study populations and larger number of segments may reveal more significant associations.

### Bone Quality and Screw Loosening

Considering that BMD based on DEXA may be erroneously elevated due to lumbar degeneration, the reduced T score is less sensitive to identify screw loosening. Therefore, some studies have begun to use HU values of vertebral bodies to predict pedicle screw loosening with good results.^6^, ^7^, ^8^ As in previous studies, our results show that patients with screw loosening have lower mean HU values in the lumbar and that low BMD is a major risk factor for screw loosening after lumbar fusion. It is well known that most pullout strength is determined by the lumbar pedicle rather than the vertebral body.^7^, ^28^, ^29^, ^30^ The bone quality surrounding the screw can affect the stability of the screw and the mechanical properties of the bone‐screw interface. Studies have shown that pedicle BMD has a greater impact on screw stability than vertebral BMD.^30^, ^31^ Therefore, preoperative assessment of local bone quality around the pedicles will help to make surgical decisions and improve the success rate of surgery and treatment results. Currently, the gold standard for assessing BMD is DEXA. However, DEXA has inherent limitations in site‐specific bone state assessment of small anatomical regions such as pedicles and is not routinely used for local bone state assessment. Studies have demonstrated that pedicle HU values tend to have a better ability to predict screw loosening than vertebral HU values.^7^, ^32^ Because studies have used different criteria and methods to determine the optimal threshold for HU values, there is still a lack of optimal HU cutting criteria to accurately cut HU values between normal and low BMD.

### MRI‐Based Bone Quality Score

Eresmann et al.^24^ proposed a new tool based on sagittal non‐contrast T1‐weighted MRI, VBQ score, to clearly differentiate healthy and osteopenic/osteoporotic patients. Early studies suggested that the increase in bone marrow adipocytes was associated with compensatory mechanisms for osteoporosis‐associated trabecular microstructural changes.^33^, ^34^ Thus, when osteoporosis occurs in bone, the trabecular portion of bone becomes more intense on T1‐weighted imaging due to fatty infiltration. VBQ score was more independent than T score and HU value in predicting screw loosening, cage subsidence, etc.^16^, ^20^, ^35^, ^36^ Therefore, MRI‐based bone quality score is a simple, effective, and reliable tool for predicting screw loosening. In recent years, it has been well known that most pull‐out strength is determined by the pedicle rather than the vertebral body.^7^, ^8^ However, several studies have explored the effect of pedicle CT HU value on screw loosening.^7^ Vertebral HU value alone is insufficient to accurately assess the risk of pedicle screw loosening. This is because the strength of screw fixation depends to a large extent on the bone condition of the pedicle. Therefore, collecting measurements of pedicle HU and vertebral HU is important for surgical planning. Based on this finding, the PBQ score was designed to specifically assess pedicle BMD. In order to measure BMD at specific sites of the pedicle, PBQ score was proposed using similar principles as VBQ score. In contrast, although VBQ also provides valuable BMD information, PBQ targets specific sites that are more directly related to screw stability and is therefore more accurate in predicting postoperative screw loosening. ROC analysis showed that PBQ, like HU, was a good predictor of screw loosening. The PBQ score optimal cutoff to differentiate patients with loosening and with non‐loosening was calculated as 3.045 with a sensitivity of 85.7% and specificity of 76.9%. PBQ score was more predictive of pedicle screw loosening than HU values (AUC: 0.751 *vs*. 0.702). Similarly, our results confirm that PBQ AUC for predicting screw loosening is significantly higher than VBQ AUC value reported in the literature.^20^ These findings indicate that PBQ score is an independent factor associated with screw loosening. It may be because it indirectly measures BMD, reflecting pedicle fat infiltration.

### Strengths and Limitations

Our study is the first to introduce a simple MRI‐based score to assess pedicle bone quality. However, our study has several limitations. Firstly, its confinement to a solitary institution, coupled with the limited scope of its participant pool, impacts the extent to which our cohort mirrors the intended demographic. Hence, additional large‐scale, randomized controlled trials with a forward‐looking approach are requisite to validate the present results. Secondly, some patients could not select pedicle ROI due to MRI scanning problems, and ROI was manually selected, which was easy to produce subjectivity. However, our study has demonstrated that PBQ measures have good inter‐rater reliability. Thirdly, more potential influencing factors such as pedicle screw size, postoperative sagittal alignment, and spine‐pelvic parameters could not be included.

## Conclusion

In conclusion, this is the first study to use MRI‐based PBQ score to predict pedicle screw loosening after posterior lumbar fusion. Our results show that PBQ score is an important predictor of lumbar pedicle screw loosening, with high PBQ score significantly associated with a higher risk of screw loosening. PBQ score was a better predictor of pedicle screw loosening after posterior lumbar fusion than HU score. Preoperative MRI‐based PBQ scores may be a promising tool to provide information on the risk of screw loosening after lumbar fusion surgery.

## Conflict of Interest Statement

The authors declare no competing interests. The authors report no conflict of interest concerning the materials or methods used in this study or the findings specified in this paper.

## Ethics Statement

This study was performed in line with the principles of the Declaration of Helsinki. Approval was granted by the Ethics Committee of West China Hospital. Written informed consent was obtained from the patients.

## Author Contribution

Lei Wang and Yueming Song contributed to the study conception and design. Material preparation was performed by Xi Yang, Haiying Fu, and Qiujiang Li. Data collection and analysis were performed by Qiujiang Li, Haiying Fu, and Huiliang Yang. The first draft of the manuscript was written by Qiujiang Li and Haiying Fu, and all authors commented on previous versions of the manuscript. All authors read and approved the final manuscript.
